# *De novo* transcriptome assembly of the grapevine phylloxera allows identification of genes differentially expressed between leaf- and root-feeding forms

**DOI:** 10.1186/s12864-016-2530-8

**Published:** 2016-03-11

**Authors:** Claude Rispe, Fabrice Legeai, Daciana Papura, Anthony Bretaudeau, Sylvie Hudaverdian, Gaël Le Trionnaire, Denis Tagu, Julie Jaquiéry, François Delmotte

**Affiliations:** Present Address: BIOEPAR, INRA, Oniris, La Chantrerie, F-44307 Nantes, France; IGEPP, INRA, F-35653 Le Rheu cedex, France; SAVE, INRA, F-33883, Villenave d’Ornon, France; IGEPP, BIPAA, INRA, Campus Beaulieu, Rennes, France; Institut National de Recherche en Informatique et en Automatique, Institut de Recherche en Informatique et Systèmes Aléatoires, Genscale, Campus Beaulieu, Rennes, France; Institut National de Recherche en Informatique et en Automatique, Institut de Recherche en Informatique et Systèmes Aléatoires, Genouest, Campus Beaulieu, Rennes, France; Present address: University of Rennes 1, UMR CNRS 6553 EcoBio, 35042, Rennes, France

**Keywords:** *Daktulosphaira vitifoliae*, Radicicole, Gallicole, Polyphenism, Oviparity, Gene duplication, Aphid, Polyphenism, *Vitis*

## Abstract

**Background:**

Grapevine phylloxera, an insect related to true aphids, is a major historic pest of viticulture only controlled through the selection of resistant rootstocks or through quarantine regulations where grapevine is cultivated own-rooted. Transcriptomic data could help understand the bases of its original life-traits, including a striking case of polyphenism, with forms feeding on roots and forms feeding in leaf-galls. Comparisons with true aphids (for which complete genomes have been sequenced) should also allow to link differences in life-traits of the two groups with changes in gene repertoires or shifts in patterns of expression.

**Results:**

We sequenced transcriptomes of the grapevine phylloxera (Illumina technology), choosing three life-stages (adults on roots or on leaf galls, and eggs) to cover a large catalogue of transcripts, and performed a *de novo* assembly. This resulted in 105,697 contigs, which were annotated: most contigs had a best blastx hit to the pea aphid (phylogenetically closest complete genome), while very few bacterial hits were recorded (except for *Probionibacterium acnes*). Coding sequences were predicted from this data set (17,372 sequences), revealing an extremely high AT-bias (at the third codon position). Differential expression (DE) analysis among root-feeding and gall-feeding showed that i) the root-feeding form displayed a much larger number of differentially expressed transcripts ii) root-feeding biased genes were enriched in some categories, for example cuticular proteins and genes associated with cell-cell signaling iii) leaf-galling-biased genes were enriched in genes associated with the nucleus and DNA-replication, suggesting a metabolism more oriented towards fast and active multiplication. We also identified a gene family with a very high expression level (copies totaling nearly 10 % of the reads) in the grapevine phylloxera (both in root and leaf galling forms), but usually expressed at very low levels in true aphids (except in sexual oviparous females). These transcripts thus appear to be associated with oviparity.

**Conclusions:**

Our study illustrated major intraspecific changes in transcriptome profiles, related with different life-styles (and the feeding on roots versus in leaf-galls). At a different scale, we could also illustrate one major shift in expression levels associated with changes in life-traits that occurred along evolution and that respectively characterize (strictly oviparous) grapevine phylloxera and (mostly viviparous) true aphids.

**Electronic supplementary material:**

The online version of this article (doi:10.1186/s12864-016-2530-8) contains supplementary material, which is available to authorized users.

## Background

Animals often display different phenotypes under different environmental conditions, a trait described as polyphenism [1]. Insects provide many examples of polyphenism, with sometimes striking differences in morphological traits and metabolism (reviewed in [2]). The extense and evolutionary maintenance of polyphenism is the result of a balance of constraints and selective forces, and reflects the ability of an organism to display flexible development systems and to fine-tune development with environmental cues [3]. It is now well established that polyphenism is characterized by marked differences in profiles of gene expression, whereas the genes or functional groups of genes affected by these genes are specific to each organism (see examples for wing polyphenism in aphids [4] or social castes in honey bees [5]).

The size of gene repertoires of each organism results from of a balance between gene duplication and gene loss, a dynamic process that could be a significant source of evolutionary novelty and adaptation [6]. Theoretical models and sequence data have been both explored to precise how and when gene duplicates can persist over time, and how they can provide a mechanism of adaptation to environmental changes [7]. As a first step into acquiring a comprehensive understanding of a striking case of polyphenism in an insect species, the grapevine phylloxera (*Daktulosphaira vitifoliae*), we have used a transcriptome sequencing approach as a way of gene expression profiling among some of its different phenotypes. We also used these data to identity gene families and get first insights on the interaction between phylogenetic aspects, in particular in gene families, and expression patterns. We have explored this interaction for gene families containing copies with the highest level expression and for a gene family showing transcripts with different patterns of morph-biased expression.

Grapevine phylloxera is an invasive pest species with worldwide economic importance [8, 9]. This insect native to North America was accidentally introduced in Europe around 1850 [10], where it became a pest of cultivated grapevine (*Vitis vinifera*) which is highly susceptible [11]. Soon after its unintended introduction, phylloxera caused the collapse of the whole European viticulture and of its economy [12]. Only after more than 30 years of research, a cure to this plague was found with the grafting of grapevine varieties on rootstocks resistant to the insects [12–14]. Phylloxera is still found in almost every wine-producing region of the world [8]. It remains a major constraint for viticulture imposing the grafting of *V. vinifera* and quarantine regulations in areas (e.g. Australia) where varieties are predominantly grown own‐rooted [15].

The life-cycle of phylloxera is special in several aspects: in particular, this insect has different forms that feed respectively on leaves and roots [16]. Individuals forming galls on leaves are called gallicoles (hereafter abbreviated as GA), and on roots, radicicoles (hereafter abbreviated as RA). Leaf-feeding forms are rare on grapevine (due to natural resistance of the plant), while the root-feeding forms are still present, even on rootstocks used for grapevine grafting [9, 11]. Given the different environmental and nutritional constraints faced by these two forms, we may expect that they are characterized by a modulation of gene expression as an adaptation to their specific conditions. The noxious effects on *Vitis vinifera* result from the root-feeding insects, which cause woundings alterating the circulation of sap and facilitating the entrance of microbial pathogens.

*D. vitifoliae* (grape phylloxera) belongs to the Phylloxeroidea, a small monophyletic superfamily of the Hemiptera closely related to the Aphidoidea (the true aphids). The Aphidoidea and the Phylloxeroidea probably diverged in the Jurassic or earlier from some aphid-like ancestor whose origin can be traced back up to about 250 My ago [17]. While not an aphid *sensu stricto*, phylloxera shares a subset of the biological traits associated with aphids, and provides an interesting model for comparative genomics among the two groups. Comparisons among the phylloxera and true aphids (for example, the pea aphid genome [18]) should indeed bring insight into the evolution of their peculiar life-traits. For example, true aphids are viviparous (except for one generation of sexual female per annual life-cycle) [19], while phylloxera is always oviparous. The two groups also differ in other aspects, such as the association with endosymbionts, sap feeding habits and digestion [20].

Hardly any genomic/transcriptomic resources exist as today for the phylloxera despite its potential economical and scientific importance. In order to identify the array of expressed genes by different forms and to provide insights into the evolution and genetic bases of specific aphid and phylloxera life-traits, we performed a first characterization of phylloxera transcriptomes. We adopted a strategy based on high throughput Illumina sequencing of cDNA, since this approach has been shown to produce high quality *de novo* transcriptomes, and is well suited for differential expression analyses [21]. We identified, annotated and compared transcripts to existing databases (including the complete pea aphid genome), allowing to discover some phylloxera-specific gene families, reconstructed a large catalogue of predicted coding sequences, and analysed patterns of nucleotidic composition. We finally performed a differential expression analysis to compare transcript abundance in leaf and root-feeding forms and found significant changes in expression among the two forms. We also identified i) a gene family that is extremely highly expressed in both morphs and appears to be associated with oviparity, illustrating major changes in expression associated with change in life-traits between phylloxera and the true aphids ii) a gene family containing two closely related copies characterized by opposite patterns of expression bias among morphs, showing a case of rapid switch in the specificity of expression following duplication.

## Methods

### Insect collection and rearing

In June 2010, an unusually high infestation of phylloxera on leaves of cv. Cabernet franc (*Vitis vinifera*) grafted on Fercal rootstock was observed at Château Couhins (Cru classé de Grave, Pessac‐Léognan, Bordeaux AOC, France). Phylloxera leaf galls were collected from this vineyard and a population was established in an insect‐proof cage in greenhouse conditions. A single leaf-galling phylloxera female was isolated from this population. Its clonal offspring (and subsequent clonal generations) were named INRA-Pcf7. Leaf-feeding insects of the INRA-Pcf7 lineage were reared on the leaves of the inter-specific hybrid Harmony which is susceptible to leaf-galling phylloxera. Harmony young plants were produced in aseptic culture conditions, planted in sterile soil within insect-proof cages. These leaf-galling insects were maintained in a growth chamber at 23 °C, 70 % humidity with a L:16/D:8 photoperiod. Root-feeding insects of the INRA-Pcf7 lineage were obtained by inoculating cv. Cabernet Sauvignon (*Vitis vinifera*) roots with leaf-galling eggs [22]. Fresh and healthy pieces 5–7 cm long of roots of Cabernet Sauvignon were washed with sterile water and placed on a wet filter paper disk inside Petri dishes sealed with parafilm. About 50 phylloxera eggs were transferred in each Petri dish and spread on these roots. The dishes were kept in plastic boxes and incubated at 23 °C, 70 % relative humidity and 24-h darkness.

Five samples were prepared in total for the sequencing experiment: two samples of leaf-feeding adult insects and two samples of root-feeding adult insects (each sample comprised 500 individuals) while the last sample was prepared with 500 eggs from root-feeding insects. Two replicates were prepared for leaf-feeding and root-feeding samples. Total RNA was extracted directly from fresh material (insects were not frozen or stored before extraction).

### RNA extraction and library preparation and sequencing

Total RNA was extracted from each of the five samples using RNeasy Kit QIAGEN for animal cells and tissues (Qiagen, Nederland), including a DNase treatment. RNA molecules longer than 200 nucleotides were bound to a silica column membrane and eluted in RNase-free water. The quantity of RNA was measured with NanoDrop® ND-1000 UV–vis Spectrophotometer with an absorbance from about 200 nm up to 350 nm. Roughly 20 μg of total RNA were obtained for each sample. The quality of the RNA samples was tested with the Agilent Bioanalyzer 2100. The intensity ratio 28S/18S after the separation of total RNA on denaturing agarose gel electrophresis was around two which corresponds to good quality samples. RNA Integrity Numbers (RIN) for the different samples ranged between 5.8 and 7.4. The cDNA libraries -Truseq, v3 chemistry, with poly-A selection, were performed according to the manufacturer’s instructions (Illumina, San Diego, CA, USA), then sequencing was done on the Hiseq2000. The five cDNA libraries were sequenced in pair-ends, with reads of 101 bp, by the GATC company (Germany). Samples were sequenced in two different runs (GA and RA on one run, while the egg sample was sequenced in another run). The raw sequence data has been deposited in the SRA division of Genbank (project accession: PRJNA294954).

### Cleaning of sequences and assembly

Analyzing the GC content and the over-representation of sequences with the fastqc software (http://www.bioinformatics.babraham.ac.uk/projects/fastqc/), we did not see any evidence of contamination, nor unexpected presence of adapters in any library. Low quality parts of the reads were trimmed from the right when the mean of phred score in a 20 bp window was below 20, with prinseq-lite (http://prinseq.sourceforge.net/). The reads larger than 20 bp after trimming were re-organized by pairs and assembled with Trinity with the jaccard_clip option to limit fusion transcripts [23] and default parameters for other options.

### Clustering of sequences

The primary assembly contained multiple contigs with closely related sequences, generally being alternative transcripts (typically differing by large indels that correspond to facultative/alternative exons, while aligning sequences were identical). To reduce this redundancy, contigs were clustered using a home-made program (available upon request) detecting contig sequences matching each other (based on a megablast search, with an identity cutoff of *p =* 0.99 over a length of at least 200 bp, and matches spanning at least 50 % of both the query and the hit). For each cluster, the longest sequence was retained. All subsequent analyses were performed on this reduced data set.

### Annotation

Contig sequences were compared by blastx (blastall program [24], version 2.2.28+) to the complete genome of the pea aphid *Acyrthosiphon pisum* (using the version 2.1 of predicted proteins, www.aphidbase.com). In parallel, a blastx search of similarity was performed against public general databases (nr, with an e-value cutoff of 1e-8). Also, completeness of the assembled transcriptome was assessed using the BUSCO software [25]: the method searches homologies to a core set of 2,712 highly conserved genes in all arthropods (we used the *Drosophila melanogaster* sequences). Finally, a blastn search was performed against potentially contaminant sequences, i.e. ribosomal RNA or mitochondrial sequences (using sequences from *A. pisum*). Then a BLAST2GO annotation helped by blastx results against nr and by an interproscan search (v4.8, against the 06/25/2013 version of interpro), was performed using the blast2go database (08/2012 version).

### Genomic features

Prediction of coding sequences (CDSs and protein sequences) was performed using the Transdecoder tool from the Trinity package [23], which uses a self-training procedure and a Hidden-Markov model approach for ORF detection. This allowed a comparison of coding sequences with the nearest completely sequenced taxon, the pea aphid. Such comparison, aimed at identifying potential orthologs and gene families that would be specific to phylloxera, was done with OrthoMCL [26]. Then the predicted CDSs sequences allowed us to evaluate compositional patterns in the phylloxera, measuring nucleotide frequencies at the three codon positions.

### Read mapping to contigs and counts of expression per library- Phylogeny of the most abundantly transcribed gene family

RNA-seq allows to capture the digital gene expression information in the form of relative read coverage. For this, the reads were aligned by pairs and by library back to the contigs with bowtie2 [27] with the non-deterministic parameter (only the best alignment is reported, or a random hit among the best if the read maps in many places). The counts of reads by contigs and library were then obtained with the samtools *idxstats* program [28]. To also get insight on the most expressed transcripts across all libraries (which could be linked with specificities of the phylloxera biology), we investigated the potential phylogenetic relationships among the corresponding genes, in particular for a 6-gene family and for their orthologs in other insect species. A phylogenetic study of this gene family was performed: the proteic alignment was obtained with T-coffee [29] then trimmed with Gblocks [30]. The phylogeny was obtained using MEGA6 [31], using a ML method and evaluating bootstrap support for nodes (1000 replicates).

### Differential expression analyses

Normalization of read counts and statistical comparison of expression of the contigs were performed using DESeq2 [32], focusing on the comparison between GA and RA libraries, for which two replicates were available. Adjusted p-values indicated levels of significance of an expression bias among the two conditions (the software using information on the variability between replicates and between conditions). The log2-fold ratio of the normalized expressions of GA (mean of the two replicates) versus RA libraries was also used to determine lists of genes that showed a morph-biased expression pattern. Differentially expressed (DE) transcripts were defined as genes with a significant difference in expression (to further decrease the risk of false positives, we selected an adjusted p-value threshold of 0.01) and a log2fold change greater than one (meaning that the contig was supported by at least twice more reads on average in one condition than in the alternative condition). An “unbiased” category was also defined (contigs for which we found an adjusted p-value >0.05 and log2fold change <1). In order to identify the putative functions of genes specifically over-expressed in each form, GO-enrichment analyses were performed by comparing the GA-biased (or RA-biased) with the “unbiased” list and also by comparing GA-biased and RA-biased genes. Multiple testing was accounted for by using the False Discovery Rate [33], with a cutoff of 0.05 to determine significant differences.

### Evolutionary rates

Putative orthologs between grapevine phylloxera (reconstructed coding sequences from this transcriptome) and pea aphid (official gene set from the complete genome) were identified by clustering protein sequences from both species (OrthoMCL). We then used a protocol for calculating evolutionary rates between 1:1 orthologs described in a recent study [34] which comprised: proteic alignment with T-coffee [29], guiding the nucleic alignment, trimming with Gblocks [30], estimation of pairwise synonymous (dS) and non-synonymous (dN) evolutionary rates using a codon-based model (Codeml, from PAML, [35]).

### Quantitative RT-PCR

For the qRT-PCR validation step, independent aphid samples were prepared and corresponding RNAs were extracted for respectively leaf-gall and root-feeding adults. Total RNAs extractions were performed according to the RNeasy Mini Kit (Qiagen) protocol. RNA samples purity and quality was checked with a Bioanalyser 2100 (Agilent) and quantified by spectrophotometery (Nanodrop Technologies). Before reverse transcription, a DNAse I treatement was performed (Sigma Aldrich). One microgram of total RNA was used for cDNA synthesis using Superscript III (ThermoScientific) and oligo dT (Promega). Primer sequences used for qRT-PCR were designed using Primer 3 software; sequences are available in Additional file 1. Quantitative PCR was performed on cDNAs products with the LightCycler 480 Real-Time PCR System using the SYBR Green I Master mix (Roche) according to the manufacturer’s instructions. A standard curve was performed for each gene using serial dilutions of cDNA products in order to assess PCR primers efficiency. A dissociation curve was performed at the end of each run in order to detect non-specific amplifications. The Q-RT-PCR experiment was done with three independent biological replicates for each condition (leaf-gall/root-feeding) and three technical replicates for each PCR point. Relative quantification was performed using the standard curve method with normalization to two invariant genes (transladolase-like and ATP-synthase-like) selected according to RNA-seq expression data (Additional file 2). Absolute measures for each of the 4 target genes *(averaged among three replicates)* were divided by the geometric average of the two invariant genes measures.

## Results

### Sequences quality, and assembly statistics

Illumina reads quality was assessed with FastQC reports, all libraries showing a good quality and satisfying parameters as for distributions of GC, qualities along the sequence, redundancy*.* Among 371,340,378 reads, only 1,672,722 (0.45 %) were removed before the assembly (Table 1). The number of reads retained after filtering and the percent of reads mapped to contigs for each library are given in Table 1. The assembly resulted in 135,861 contigs, which were reduced to 105,697 sequences after clustering of near identical sequences (Table 2) - all subsequent results concern this reduced set of contigs. With 14,420 contigs longer than 1,000 bp, and given the distribution of contig sizes we may consider that the assembly was of good quality and generated a large number of relatively long contigs, with likely a high proportion of contigs covering full length transcripts.

**Table 1:** Read counts (number of read pairs) per library, generated by Illumina RNAseq, and percentage of reads mapped on contigs

Libraries		# of read pairs	% mapped reads
Gallicoles, replicate 1	GA1	93940358	74.0 %
Gallicoles, replicate 2	GA2	50515816	94.0 %
Radicicoles, replicate 1	RA1	88938890	94.0 %
Radicicoles, replicate 2	RA2	95805560	93.8 %
Eggs	EGG	42139754	93.9 %

**Table 2:** Statistics from the *de novo* transcriptome assembly of the grapevine phylloxera. Except for the number of contigs, all statistics are given in numbers of base pairs

	Assembly statistics
Number of contigs	105,697
Total size of contigs	66,819,737
Shortest contig	201
Longest contig	24,038
Number of contigs > 500	32,824
Number of contigs > 1 K	14,420
Number of contigs > 10 K	59
Mean contig size	632
Median contig size	346
N50 contig length	936

### Assessment of completeness and annotation

We assessed completeness using the BUSCO method, based on a set of 2,712 conserved genes among nearly all arthropod genome. We found that 95.0 % of those genes (*D. melanogaster* sequences) had a hit in our assembled transcriptome. Moreover, the average percentage of “recovery” (length of the target gene that is matched) was 75.6 %: given that a gene is considered as tentatively complete at 80 %, these statistics together suggest a high completeness of our assembly. The Blastx comparison with nr showed that 15,433 (roughly 15 %) contigs had a hit (Table 3). We noted however that 66 % of contigs longer than 1,000 bp had a hit while the bulk of no-hit contigs corresponded to the smaller classes of size (Additional file 3). For a large majority of cases (74.7 %), the first hit was from the pea aphid. A significant minority had however a hit in another insect species (including other aphids), suggesting either that these genes have been lost in an ancestor (true aphid-like) of the pea aphid or that the gene has not been correctly annotated in that species. Reflecting phylogeny, the mean percentage of identity was higher for aphid hits (66.7 % on average for pea aphid and 71.6 % for other aphid matches) than between phylloxera and any other insect species. However, high identity hits to non-insect organisms were also recorded for three additional taxa: the bacteria *Propionibacterium acnes* (*n =* 135) – see discussion, *Vitis* sp. (*n =* 26) -possibly corresponding to host plant transcripts- and a grapevine virus (*n =* 49). A GO annotation was found for 6,657 contigs, a low percentage of all contigs but representing 40 % of contigs having a blast hit on nr.

**Table 3:** Results of the blastx comparisons between contigs from the grapevine phylloxera transcriptome and proteins from the nr bank, ordered by the number of contigs which have a given species as best hit. In some cases, species of a same genus (e.g. *Drosophila*) or of a same higher order taxonomical group (e.g. all sequences from true aphids – Aphidoidea - except the pea aphid: *Aphis* sp., *Myzus persicae*, *Rhopalosiphum* sp., *Toxoptera citricida*) were binned

best hit species	Group	# contigs	Mean % identity
Acyrthosiphon pisum	Insect (Hemiptera)	11534	66.7
Tribolium castaneum	Insect (Coleoptera)	536	43.1
Camponotus floridanus	Insect (Hymenoptera)	209	51.4
Drosophila sp.	Insect (Diptera)	158	45.9
Hydra magnipapillata	Cnidaria	147	49.4
Propionibacterium acnes	Bacteria	135	97.1
Danaus plexippus	Insect (Lepidoptera)	109	44.8
Acromyrmex echinatior	Insect (Hymenoptera)	106	53.2
Pediculus humanus corporis	Insect (Phtyraptera)	100	51.2
Strongylocentrotus purpuratus	Echinodermata	93	45.2
Megachile rotundata	Insect (Hymenoptera)	82	48.1
Harpegnathos saltator	Insect (Hymenoptera)	77	49.2
Bombyx mori	Insect (Diptera)	75	43.7
Metaseiulus occidentalis	Insect (Hymenoptera)	70	48.3
Malus sp.	Plant	66	46.3
Nasonia vitripennis	Insect (Hymenoptera)	64	51.3
other aphid species	Insect (Hemiptera)	58	71.6
Aedes aegypti	Insect (Diptera)	50	43.5
Ostreococcus lucimarinus CCE9901	Plant	49	55.7
Grapevine rupestris stem virus	Plant virus	49	96.3
Vitis sp.	Plant	26	91.2

The mean % identity (of the first match –or hsp) is given. Results are given for the top 20 numbers of contigs, and also for genes from *Vitis* (host-plant of phylloxera)

### Gene repertoires, identification of gene families unique to the phylloxera

Protein prediction in the assembled contigs resulted in 17,372 predicted proteins, of which 12,617 were predicted complete. We examined the relationships between this set of proteins and the nearest genome (that of the pea aphid). Our aim was to identify gene families within and between species, as a first evaluation of their respective dynamics of duplication/gene loss. We found 7,103 one to one families, putatively corresponding to orthologs among the two species (Table 4). Additionally, 1,279 families corresponded to genes with several gene copies in at least one of the two species (“many to many” or “many to one” families). The number of genes in these families was generally higher in *A. pisum* than in the phylloxera, which is expected given that the *A. pisum* and phylloxera data are respectively based on a complete genome and on a *de novo* transcriptome. A significant number of families (683), totaling 3,047 genes, were found to be specific to phylloxera (Table 4). Since our comparison involved only two species (grapevine phylloxera and the pea aphid) this does not imply that all of these genes are “specific” in a broader sense (some of these genes could be similar to other genes from different organisms). However, we found that a majority of these genes (78.2 %) had not hit to the nr database. This is the case of the two largest phylloxera-specific families which comprised 69 and 63 peptides respectively. Therefore gene families truly specific of *D. vitifoliae* appear to represent a significant fraction of this transcriptome-based gene collection.

**Table 4:** Gene family identification among predicted proteins from the grapevine phylloxera (transcriptome-based) and proteins from the pea aphid genome (official gene set, version 2.1), using OrthoMCL

	#families	#genes, pea aphid	#genes, phylloxera
Many to Many	137	988	379
Many (pea paphid) to one (phylloxera)	935	3438	935
Many (phylloxera) to one (pea paphid)	207	207	450
One to one	7103	7103	7103
Pea aphid specific families	3702	14,995	0
Phylloxera specific families	683	0	3047
Phylloxera specific singletons	-	-	5458
Pea aphid specific singletons	-	10,259	-
Total # genes per species	-	36,990	17,372

The first column precises the different categories of homology relationships: gene families may contain several copies in both species which are co-orthologs (“Many to Many”, all these genes being co-orthologs), several copies in one species ortholog to only one copy in the other species (“Many to one”), only one copy in each species (1:1 orthology, or “One to one”), or yet may contain copies in only one species (“Pea aphid-specific“ or “Phylloxera-specific”). Finally, genes from one species not found in gene families and without any ortholog in the other species are “Singletons”. The numbers of families of each type are given, along with the number of predicted genes from each species for each category

**Fig. 1:**
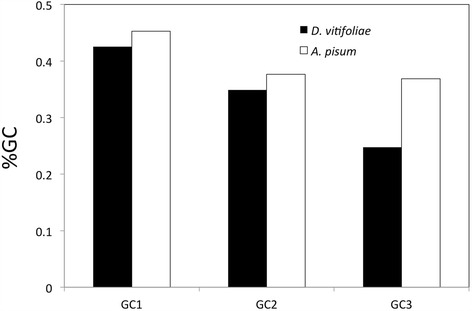
Mean percentage of GC at the first, second and third codon positions for pea aphid (*A. pisum*) – version 2.1 of the official gene set (*n =* 36,990 CDSs) [18]- and for the grapevine phylloxera (*D. vitifoliae*) *de novo* transcriptome (*n =* 12,617 predicted complete CDSs)

Finally, the compositional patterns of the predicted complete CDSs showed a strong shift between phylloxera and the pea aphid (Fig. 1). The pea aphid has been found to have a high AT content, particularly at the third codon position compared to other insect genomes [21]. Genes of grapevine phylloxera were found to be even more AT rich than in the pea aphid (with 24.8 % vs 36.9 % at the third positions, in the grapevine phylloxera and pea aphid CDSs respectively).

### Identification of very abundant transcripts, phylogenetic study of their gene family

**Fig. 2:**
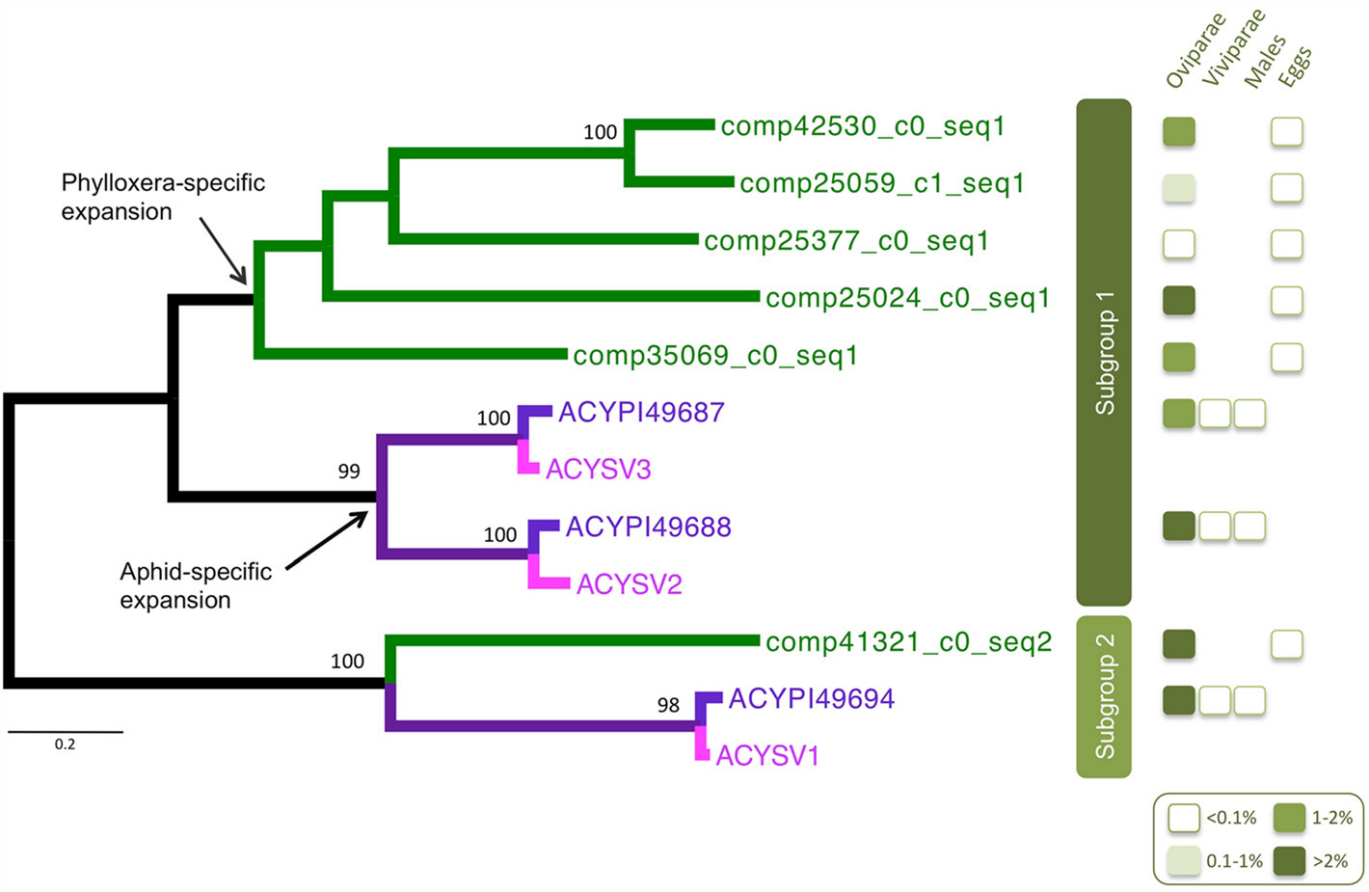
ML phylogenetic tree (JTT model, with MEGA6) of the gene family containing some of the most highly abundant transcripts in the phylloxera transcriptome (both for radicicoles and gallicoles libraries). The phylogeny includes 6 identified copies from the phylloxera transcriptome (“comp” prefix), three homologs from the pea aphid genome (“ACYPI” prefix) and three homologs from a transcriptome of *Acyrthosiphon svalbardicum* (“ASV” prefix). Grapevine phylloxera specific branches are colored in green, while branches for true aphids are in purple (ancestral branches), blue (*A. pisum*) or pink (*A. svalbardicum*). The two subgroups would correspond to an ancestral duplication (pre-dating the split between true aphids and phylloxera), while further lineage-specific duplications would have occurred in subgroup 1, as pointed by arrows at two nodes. Bootstrap values (above 80) are given at the nodes. On the right, class percentage of the mapped reads for different libraries (whole individuals, different morphs) – for detailed counts, see Table 4

We examined the characteristics of the most abundant transcripts, whether or not they showed a biased expression among morphs. In particular, the most supported transcript in RA represented more than 5 % of the mapped reads (Table 5). We checked CDS predictions for that transcript and for potential related sequences in the transcriptome (paralogs). We found a total of six gene copies with predicted full length peptide (sequence length of *ca* 470 amino-acids). Several of these copies have a high expression in GA and RA (totaling more than 1 % of the mapped reads) – but not in the egg library. This gene family for which we predicted a signal peptide (SignalP analysis [36]) showed no similarity outside aphids. However, these genes had hits to three weakly similar copies in the pea aphid – all hypothetical proteins - and also to three transcripts from the transcriptome of *Acyrthosiphon svalbardicum* (a transcriptome recently obtained by our group for sexual oviparae females, unpublished data). The phylogenetic analysis of this family shows two sub-groups (corresponding to an ancient duplication, preceding the phylloxera-true aphid divergence) – Fig. 2. In sub-group 1, lineage-specific duplications further occurred either in true aphids or in the phylloxera lineage. Expression data for the pea aphid [37] show that the three copies in this species are highly specific to oviparae (these copies totalize 10 % of the mapped reads in that morph while they are expressed at very low levels in males or viviparous asexual females) – Fig. 2, Table 5.

**Table 5:** Percentages of read counts for transcripts in a gene family (number of reads divided by the total of mapped reads in each library, averaged among replicates) comprising the most abundant transcript in gallicoles (GA) and radicicoles (RA), comp25024_c0_seq1

Phylloxera transcripts	Gallicoles (oviparae)	Radicicoles (oviparae)	Eggs
comp25024_c0_s eq1	3.80	5.49	0.02
comp41321_c0_seq2	1.94	1.98	0.01
comp42530_c0_seq 1	1.10	2.02	0.01
comp35069_c0_s eq1	0.68	1.61	0.00
comp25059_c 1_seq1	0.33	0.34	0.00
comp25377_c0_seq1	0.002	0.001	0.00
Total	7.86	11.43	0.04
Pea aphid genes	Sexual females (oviparae)	Asexual females (viviparae)	Males
ACYPI49687	1.84	0.001	0.00
ACYPI49688	4.31	0.02	0.02
ACYPI49694	4.44	0.003	0.00
Total	10.59	0.02	0.02

For the grapevine phylloxera percentages of read counts in GA, RA and egg libraries are given for the six paralogs identified. For the pea aphid, percentages of read counts in libraries obtained from three different reproductive morphs (whole bodies of oviparous sexual females, viviparous asexual females, males) are given for the three paralogs identified [21]

### Differential expression analyses

**Fig. 3:**
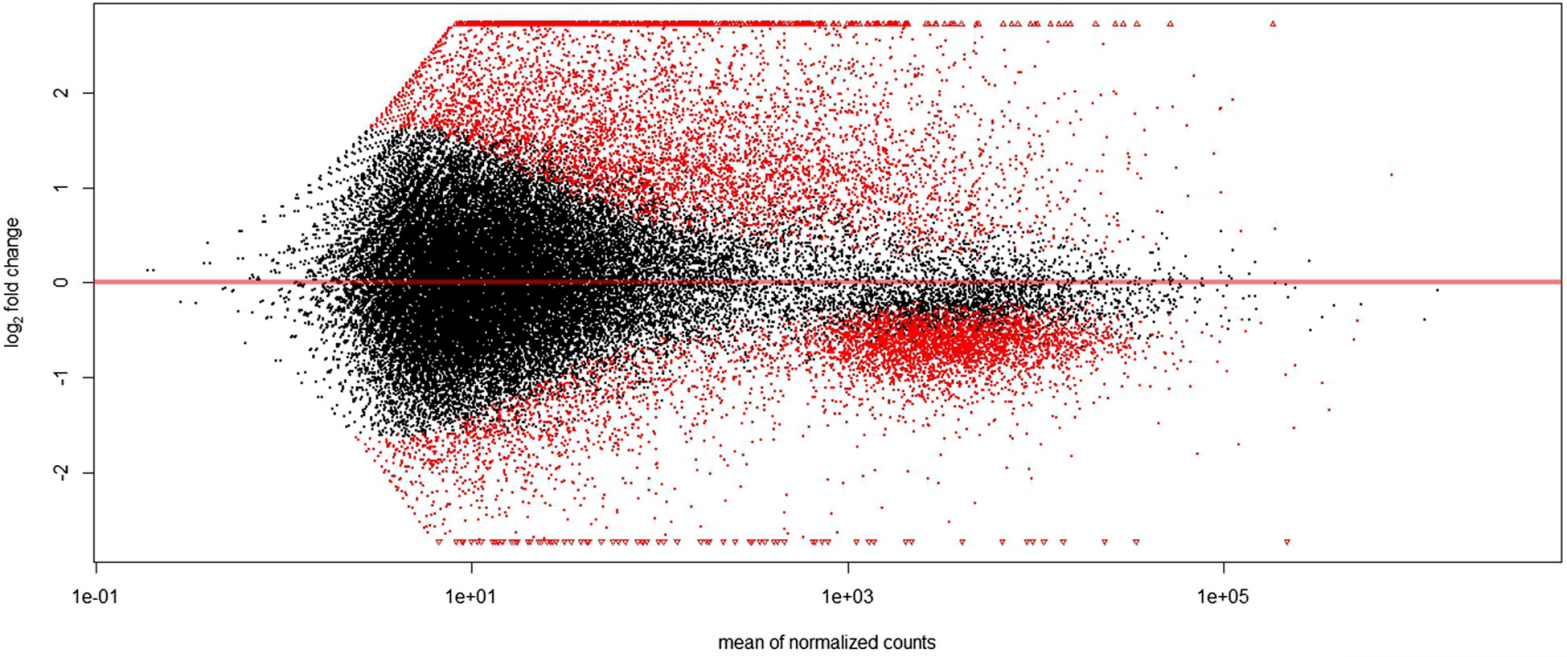
Result from the differential expression analysis (with Deseq2) between leaf- and root-feeding morphs of phylloxera. The figure shows log2fold changes (radicicoles/gallicoles) in expression for each contig in y-axis and mean normalized read counts in x-axis. Above the x-axis, genes that are more expressed in the root-feeding morph (radicicoles), below that line, genes that are more expressed in the leaf-feeding morph (gallicoles). In red, statistically significant DE (differentially expressed) contigs among the two feeding conditions

The change in expression level among the two conditions (log2fold change), for every contig, is represented in Fig. 3. This figure shows that the distribution of genes that are more expressed in RA or GA were markedly different. GA-biased genes tended to represent abundant transcripts, with a moderate ratio increase of expression compared to RA. By contrast, RA-biased represented less abundant transcripts overall, with a high ratio increase of expression compared to GA. If we consider genes that are significantly biased at the *p =* 0.01 level and show a log2fold of absolute value > 1 (meaning that absolute number of reads in normalized counts changes at least twofold among conditions), many more genes appeared to be RA-biased (*n =* 3,566) than GA-biased (*n =* 883). Besides, many more genes had only support in the RA condition (*n =* 11,077) than genes that had only support in the GA condition (*n =* 4,054).

**Fig. 4:**
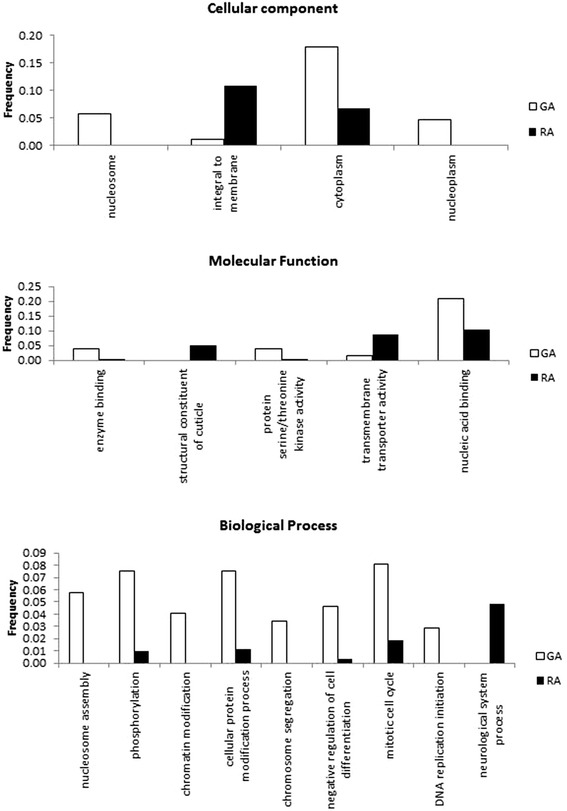
Gene ontology enrichment analysis (Fisher test, False Discovery Rate < 0.05) between genes that are significantly over-expressed in gallicoles (RA) or in radicicoles (RA). In y-axis, frequency of terms in GA- and RA-over-expressed transcripts

To get insight into the functional characteristics of differentially-expressed (DE) transcripts (as defined above), we compared their GO annotation using enrichment-tests based on a Fisher–test with a False Discovery rate or FDR of 0.05 (Fig. 4, which details the GO-terms statistically more associated with respectively RA-biased or GA-biased transcripts). For the Molecular function category, RA-biased were much richer in genes annotated as “structural component of the cuticle” (of note, the same genes were also often associated with the Cellular component term “integral to membrane”). To further confirm this we analysed the distribution of log2fold ratios of expression between RA and GA for all 60 contigs annotated as “cuticular protein” and found this distribution was heavily shifted to positive ratios (58/60 contigs having higher expression in RA than GA, Additional file 4). RA-biased contigs were also enriched for transmembrane/transporter activity, and neurological system process (genes often also associated with cell-cell signaling). On the other hand, GA-biased genes were much more frequently associated with the nucleosome and with the cytoplasm, DNA replication, and with protein serine kinase activity –several of the top expressed gene in GA and with high log2fold ratio being serine proteases. Information for each contig (expression measures in the different libraries, GO annotation, blastx annotation, OMCL clustering) is summarized in Additional file 5.

#### qPCR validation

**Fig. 5:**
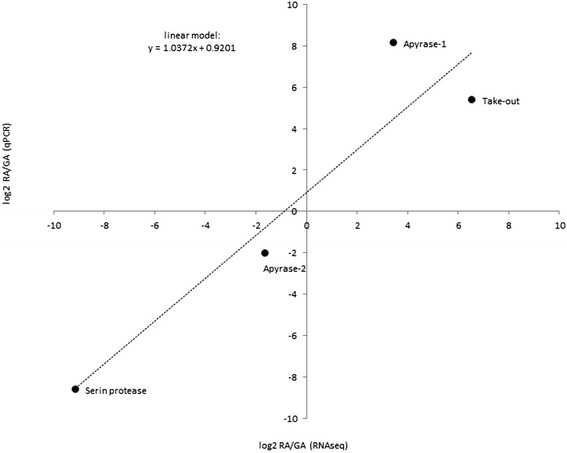
Correlation between foldchanges of expression (log2-transformed) between root-feeding and leaf-gall feeding forms, measured by RNAseq (x-axis) and qPCR (y-axis) for four target genes. The correlation coefficient was 0.938 and the linear regression equation is displayed on the graph

To validate RNAseq measures of expression and subsequent identification of statistically DE genes among leaf-gall and root-feeding forms, we independently measured expression using qPCR. For that purpose, we selected 6 genes, based on RNAseq results. Two genes had been identified as significantly more expressed in root feeding forms (apyrase-1, take out), two other genes had been identified as significantly more expressed in leaf-gall feeding forms (apyrase-2, trypsin-like serine protease) while the last two genes, transaldolase-like and ATP-synthase-like showed a constant expression level (respectively at moderate and high levels) and were used to normalize qPCR measures of expression. We observed a strong correlation (correlation coefficient of 0.938) between the ratios of expression among the two morphs measured with the two methods, RNAseq and qPCR (Fig. 5). The genes over-expressed in either leaf-gall-feding of root-feeding forms showed the same patterns with both approaches, which gives a strong support to RNAseq based counts of expression.

### Evolutionary rates

**Fig. 6:**
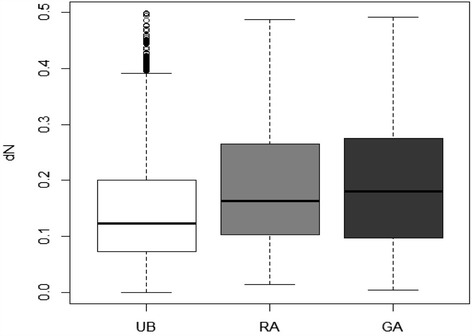
Non-synonymous rates (dN) for unbiased (UB, *n =* 25,683), GA-biased (GA, *n =* 1,125) and RA-biased (RA, *n =* 4,485) genes: boxplot of pairwise estimates of dN between putative orthologous sequences of grapevine phylloxera and of the pea aphid

The non-synonymous and synonymous rates and their ratios were estimated for 7103 putative orthologs between the pea aphid and grapevine phylloxera. Synonymous rates were found to be generally largely above unity, suggesting saturation, which could be expected given the phylogenetic distance between phylloxera and the true aphids. We therefore did not consider estimates of dS or the dN/dS ratios, but rather focused on the dN rates, comparing evolutionary rates for genes belonging to different categories of expression. GA-biased showed the highest non-synonymous rates, followed by RA-biased genes, then by genes with an unbiased expression (Fig. 6).

## Discussion

The aim of this study was to develop new sequence resources for the grapevine phylloxera (*D. vitifoliae*), an organism which is both a major pest for viticulture and an important model for comparative genomics studies of plant sucking insects. By sequencing RNA libraries of different morphs, we assembled and explored the phylloxera transcriptome, building a collection of coding sequences and comparing these sequences with the complete genome of a true aphid. We could by the way evaluate the presence of potential phylloxera-associated bacteria species, and finally compared the expression patterns of two widely different feeding conditions and environments.

By combining Illumina sequenced RNA libraries of different forms we have been able to reconstruct the first *de novo* transcriptome of grapevine phylloxera, confirming that is approach is a very useful tool for generating large-scale information on the coding sequences of non-model organisms (for recent examples, [38–40]). We indeed obtained 105,697 different transcripts, for which we predicted 17,372 proteins of which 12,617 being complete predicted proteins*.* RNA-seq approaches may fail to record sequences from very rare transcripts. Still, such high number of predicted genes is in the same range than the expected number of proteins identified in completely sequenced and well annotated genomes from different insect species (e.g. 13,953 for *Drosophila melanogaster*, release 6.01). Nonetheless, a much higher number of predicted proteins was proposed for the true aphid *A. pisum* (36,990; [41]), which along with daphnia [42], has an exceptionally duplication-rich genome for an arthropod. Although the exact timing of the vast gene expansion characterizing the pea aphid lineage is yet to be determined, the distribution of genetic distances among paralogs of *A. pisum* suggested that most duplications took place relatively recently in the evolution of aphids, and probably well after the divergence between true aphids and Phylloxeroidea [18]. Therefore, we may expect that the number of different proteins for grapevine phylloxera would be closer to the average of other insect genomes, and thus that the transcriptome based predicted genes we obtained should represent a majority of the coding genome.

As a first attempt to annotate and characterize this assembled phylloxera transcriptome, we analysed blast hits to the nr database. Around 75 % of the 15,455 contigs with a hit matched to the pea aphid, while the next top species collected only 3 % of the hits. This reflects the much closer phylogenetic relationship between phylloxera and true aphids, compared to any other known genome. Another facet of this investigation was the potential to discover genes specific to the focus organism, and which could play a role in its specific adaptations. It is difficult to make any inference on single-copy gene with no known similarity to any other organism (because for example they may represent over-predicted open reading frames). But we hypothesize that families of predicted genes specific to phylloxera would be more likely to represent true genes and to be associated with some of the specific life-traits of this species. The OrthoMCL conducted in this study, comparing the pea aphid genome and phylloxera proteins, allowed to identify 683 families totaling 3047 genes unique to phylloxera. Further, most of these genes had no similarity to any known protein. For example, the two largest gene families comprised each up to 60 genes. Analysing the complete peptides in these families, we found the presence of a signal peptide (according to SignalP prediction), which suggest they could be secreted proteins, possibly playing a role in nutrition or host plant interaction [43].

We also analyzed the specificities of this grapevine phylloxera transcriptome in terms of global expression levels, comparing to available data for the pea aphid for which extensive transcriptomics data have been produced. We focused on the contigs with the highest level of support, and thus the transcripts which appear to be the most highly expressed, and examined the nature of genes predicted for these contigs. Several of the top contigs appeared to represent a six-member gene family in the phylloxera, with similarity to another three-member gene family in the pea aphid. In total, the six genes for phylloxera comprise roughly 10 % of the sequenced reads both in radicioles and gallicoles, which are both oviparous asexual females, strict oviparity being a characteristic of this group (Phylloxeroidea). Analyzing expression of homologous genes from the pea aphid, we also found a similar high percentage of the total reads in one specific morph, the sexual oviparous females. But transcription levels were very low either in asexual females, the viviparous morph which largely dominates the life-cycle of *A. pisum*, or in males. Also, we recently sequenced a transcriptome for sexual oviparous females of *Acyrthosiphon svalbardicum* (unpublished data) and found an abundant level of the homologous genes in that data set. There is therefore a strong relationship between oviparity and a high level of expression of these transcripts. It is not clear to infer a function for these genes since no similarity was found outside the group of aphids. This is possibly related with the fact that members of this gene family are clearly very fast-evolving sequences, as deduced from the low score of alignments and long branch lengths of the tree. We may however hypothesize that these proteins are linked both in phylloxera and true aphids with the production of eggs, sexually or asexually, possibly playing a role of nutritional reserve. Our results therefore illustrate major shifts in transcription levels occurring along evolution and accompanying shifts in biological traits (here a transition from oviparity to viviparity in the ancestor in true aphids). Comparable shifts in expression have identified in cave fish [44] and associated with adaptation to obscurity and reduced eye function.

The large collection of predicted coding sequences also allowed us to study the distribution of the GC content, distinguishing the different codon positions and comparing these statistics with the pea aphid. The pea aphid genome has been found to have a relatively high AT content (in particular at the third codon position with %GC3 = 36.9) compared to other insect genomes. Similar GC content was recorded for coding sequences from other species of Aphididae [18, 45]. The GC content at third codon position is typically most subject to change among organisms, as it often concerns synonymous sites. We found that the phylloxera CDSs are even richer in AT (%GC3 = 24.8), which seems to place it among the most AT-rich genomes among arthropods. A strong compositional shift has therefore occurred along the divergence between true aphids and the ancestor of phylloxera.

Nearly all members of the true aphids (Aphidoidea) possess endosymbiotic primary symbiotic bacteria localized in specialized cells (mycetocytes) which are localized next to the gut lumen [46]. These endosymbiotic bacteria (*Buchnera*) provide their host with essential amino acids that are not synthesized by the insect. Early studies [47] explored the possibility of symbionts in the grape phylloxera and identified structures which were thought to harbour symbionts. But this interpretation was contradicted by other authors [48, 49] and Buchner [46] excluded the existence of *Buchnera aphidicola* in *D. vitifoliae* in his work on aphid symbiosis. Recently, it has confirmed the absence of *Buchnera* in *D. vitifoliae* using molecular genetic approaches. However, this author identified bacteria closely related to *Pantoea agglomerans* in parthenogenetic individuals and their eggs and leaf gall tissue for several grapevine phylloxera populations investigated. These bacteria were culturable on simple media suggesting no obligate relation with the host [50]. Also, for another Phylloxera species associated with hickory trees, *Pantoea agglomerans* was found in some but not all populations, suggesting that the association is not strict [51]. A recent study demonstrated the presence of *Pantoea* sp. in vineyards soil and the bark of grapevine indicating that these bacteria could have a free-living lifestyle or be associated with the plant [52]. In our study, we found few bacterial transcripts overall, with one exception (see below), and no contig of our assembled phylloxera transcriptome matched to *Pantoea*. We must remain cautious about the significance of this negative finding, given that our purification protocol using poly-A selection may have hindered detection of bacterial transcripts. Further studies with different protocols would be necessary to better evaluate the prevalence of *P. agglomerans* in that insect species.

However, we found abundant traces of transcripts (135 different contigs) from a bacteria closely related with *Propionibacterium acnes*, normally known as a human opportunistic pathogen. Although we first thought of contamination to explain this presence, a recent study [53] has since brought biological and phylogenetic evidences for a recent association of this human pathogen with grapevine (*V. vinifera*). Fluorescent in-situ hybridization helped localize *P. acnes* in the bark, the xylem, and pith tissues of grapevine. The inability to cultivate any of the strains isolated suggested a symbiotic interaction with the host plant. Our result could therefore be explained by the presence of *P. acnes* in the plant sap ingested by the insects feeding in xylem (a specificity of phylloxera, by contrast with true aphids that feed on phloem).

We have been able to identify many genes with a bias in expression among two forms with contrasted feeding habits, e.g. the forms feeding on roots and leaves of grapevine. First, a much greater number of genes appeared to be expressed in the radicicole form than in the gallicole form. This might be consistent with the fact that gallicoles live in a protected environment, where some functions are less essential. For example, one of the striking differences concerned cuticular proteins, which were nearly systematically more expressed in RA than in GA librairies. This fits well with our own observations of the different morphs, gallicoles showing a thinner and more pale tegument than radicicole forms. Also, the literature reports that gallicole forms are distinct from radicicoles with respect to reproduction [49, 54]. Gallicole forms indeed are highly fecund compared to radicicoles. This might explain the much higher importance of functions associated with DNA replication in gallicoles. Another important difference concerns genes that would be involved in neurological processes (a category which we found to be largely over-lapping with another GO annotation term, cell-cell signaling). These annotations are absent from the GA-biased set of genes, but well represented in the RA-biased genes. Possibly, more interactions with the environment are imposed on individuals having to feed underground, than on individuals protected in the leaf galls. Of note, two of the genes with the most negative log2fold change (much higher expression in GA compared to RA) and very high absolute level of expression in GA are annotated as serine protease, while a third gene in that category has similarity with Megourin, a peptide identified in a true aphid species, *Megoura viciae*. In insects, serine proteases have been notably associated in digestion and defense responses towards both microbial and parasitoid wasp invaders [55]. Megourin, first identified in the vetch aphid *Megoura viciae* (P. Bulet *et al.*, unpublished data) could be associated with innate immunity [39]. On the other hand, several of the genes with the most positive log2fold change (much higher expression in RA compared to GA) and very high absolute level of expression in RA are annotated as cytochrome P450-like. This ubiquist gene family is often associated in animals with development or with the metabolism of toxic compounds. At present, there is still no easy explanation for these differences between GA and RA with respect to potentially immune-related or detoxification systems. Another interesting result of this study is that paralogous sequences (gene copies that derive from duplication) may display sharp differences in expression specificity among conditions. We detected this at least for a pair of transcripts corresponding to two different copies of a gene similar to apyrase, an enzyme known to hydrolyse ATP (for phylloxera, these genes were named apyrase-1 and apyrase-2). These two copies were among the four target genes selected for the qPCR validation of RNAseq measures of expression. We found indeed that apyrase-1 and apyrase-2 which are 94.9 % nucleic identity and 93.54 % proteic identity -such high identity suggesting that they derive from a relatively recent duplication event- are respectively specific to leaf-gall-feeding and root-feeding forms. This provides an example of specialization of gene copies following duplication [56]. Finally, it is important to consider that in our experimental design, leaf-gall and root-feeding were exposed to different plant species, to closely reflect the field situation. Indeed, GA are the dominating form on the wild American species of *Vitis*, while RA essentially multiply on the roots of the cultivated grapevine. Therefore, the observed patterns of differential expression might have been explained by a combination of intrinsic differences among morphs and of host plant effects.

A final point of our study is the clear difference in evolutionary rates among genes with a biased expression and genes with an unbiased expression, the former showing faster-evolving protein sequences. It has been found that restricted gene expression breadth results in accelerated evolution, as has previously been demonstrated with studies on sex-specific and tissue-specific gene expression [57, 58]. Insect polyphenism is determined by alternative gene expression profiles meaning that different subsets of genes contribute to the different morphs (e.g. [4, 59, 60]). Therefore, this can theoretically affect their rate of evolution, morph-biased gene showing relaxed purifying selection [61]. A recent study on *A. pisum* [34] demonstrated that morph-biased genes exhibit faster rates of evolution (in terms of dN/dS) relative to unbiased genes. This pattern in *A. pisum* was particularly noticeable when differential expression rose to 5-fold or higher. These highly morph-biased genes may be functionally relevant for the morphs yet simultaneously be rapidly evolving in a potentially non-adaptive manner. The results on *D. vitifoliae* obtained here confirm this view. These results join the growing body of work showing that morph-biased genes evolving more quickly than ubiquitously expressed genes.

## Conclusions

We have identified important differences among transcription levels of two forms of the grapevine phylloxera, suggesting that the two feeding conditions impose very different constraints and metabolisms. At a different scale, we have also identified one major shift in expression between grape vine phylloxera and true aphids, concerning a gene family for which very high expression levels seem to be associated with oviparity. More extensive work will be needed to clarify the functional signification of these changes of expression, and how they may be associated with specific life-traits. Also, more data will help determine the interaction between shifts in expression levels and the possibly different evolutionary pressures on the sequences of genes with different breadth of expression.

## Additional files

Additional file 1: Primer sequences used for qRT-PCR. (XLSX 9 kb) Additional file 2: Heatmap of adjusted expression levels (reads per million, or rpm, values displayed in the cells) evaluated through RNAseq, for the six genes used for qPCR validation of gene expression. Columns represent RNAseq libraries, with two samples from gall-feeding forms (GA) and two samples from leaf-feeding forms (RA). Lines correspond to the six selected genes. Genes are ordered from top to bottom from a lesser mean ratio between RA and GA to a higher ratio. The first two genes were found statistically GA-biased while the bottom two genes were RA-biased. The two genes boxed were not DE and showed minimal variation among all samples: these two genes were chosen to normalize qPCR measurements. (JPEG 95 kb) Additional file 3: Distribution of contig sizes and percentages of contigs with a hit in the nr database for each size bin. Contig sizes in bp, on the left y-axis, with a logarithmic scale; size bins of 200 bp intervals. (JPG 41 kb) Additional file 4: Distribution of log2fold ratios of expression (RA/GA) for 60 contigs annotated as “cuticular protein”. (JPG 18 kb) Additional file 5: Expression statistics and annotation information for each contig. Contig name, size (in bp), raw number of reads for each library (GA1, GA2, RA1, RA2), GO annotation (first line of the BLAST2GO output), blastx on nr (accession of the first hit, percent of identity, description), OMCL clustering in the pairwise comparison with the pea aphid genome (number of genes in the gene family, number of species, number of pea aphid genes and number of phylloxera genes). (ODS 4225 kb)

## Abbreviations

DE: differential expression/differently expressed
GA: gallicoles
GO: gene ontology
RA: radicicoles
