# Supplementary figures and images for: De novo transcriptome assembly of the grapevine phylloxera allows identification of genes differentially expressed between leaf- and root-feeding forms
Source: BMC Genomics. 2016 Mar 11;17:219. doi: 10.1186/s12864-016-2530-8 (PMC4787006; doi:10.1186/s12864-016-2530-8)

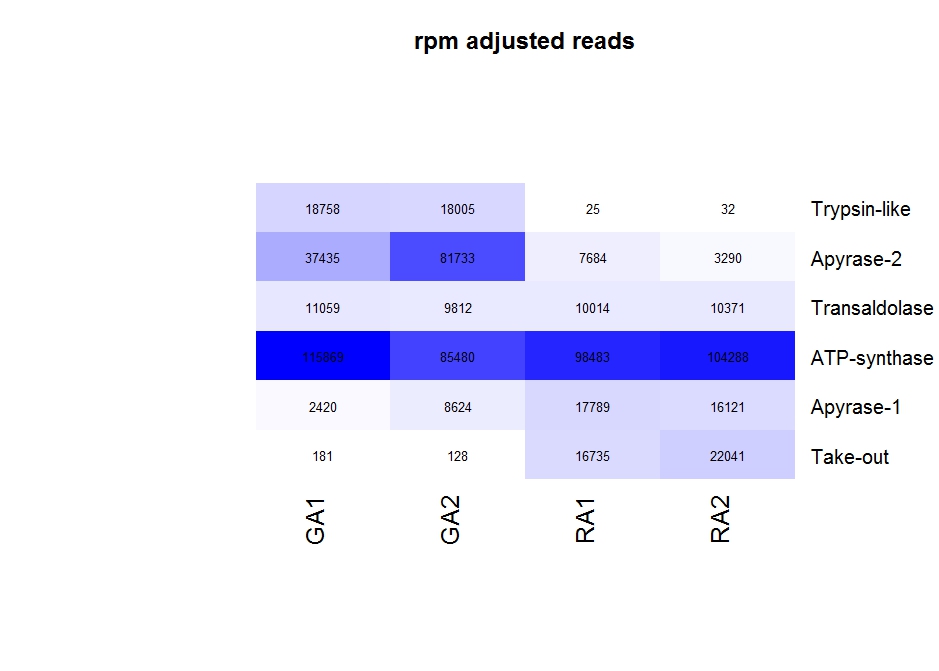

Supplement: Additional file 2: — Heatmap of adjusted expression levels (reads per million, or rpm, values displayed in the cells) evaluated through RNAseq, for the six genes used for qPCR validation of gene expression. Columns represent RNAseq libraries, with two samples from gall-feeding forms (GA) and two samples from leaf-feeding forms (RA). Lines correspond to the six selected genes. Genes are ordered from top to bottom from a lesser mean ratio between RA and GA to a higher ratio. The first two genes were found statistically GA-biased while the bottom two genes were RA-biased. The two genes boxed were not DE and showed minimal variation among all samples: these two genes were chosen to normalize qPCR measurements. (JPEG 95 kb) [file 12864_2016_2530_MOESM2_ESM.jpeg]

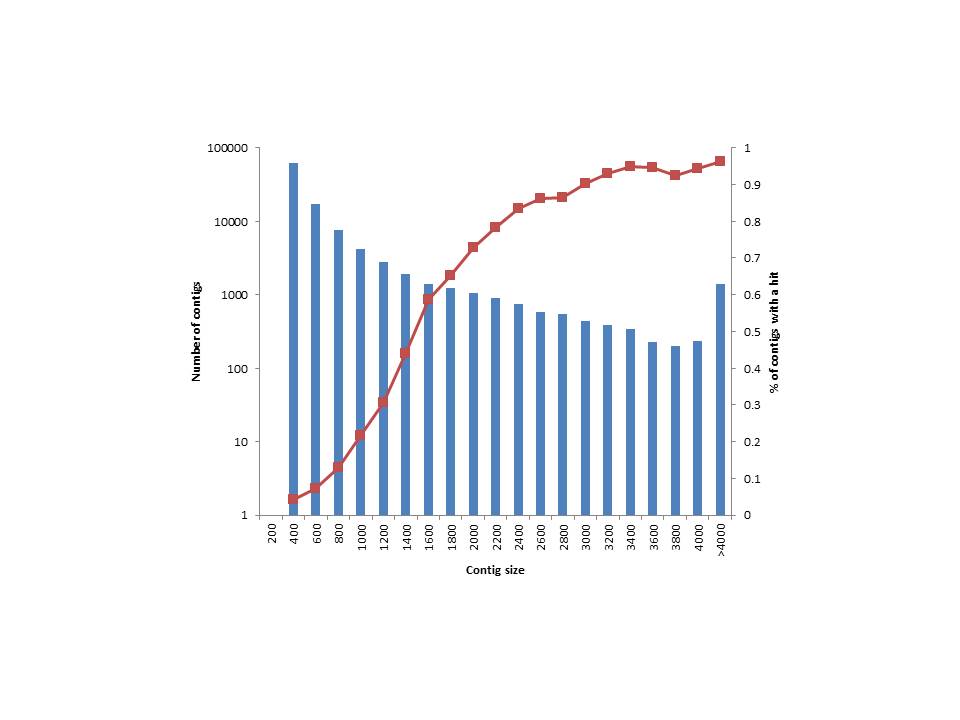

Supplement: Additional file 3: — Distribution of contig sizes and percentages of contigs with a hit in the nr database for each size bin. Contig sizes in bp, on the left y-axis, with a logarithmic scale; size bins of 200 bp intervals. (JPG 41 kb) [file 12864_2016_2530_MOESM3_ESM.jpg]

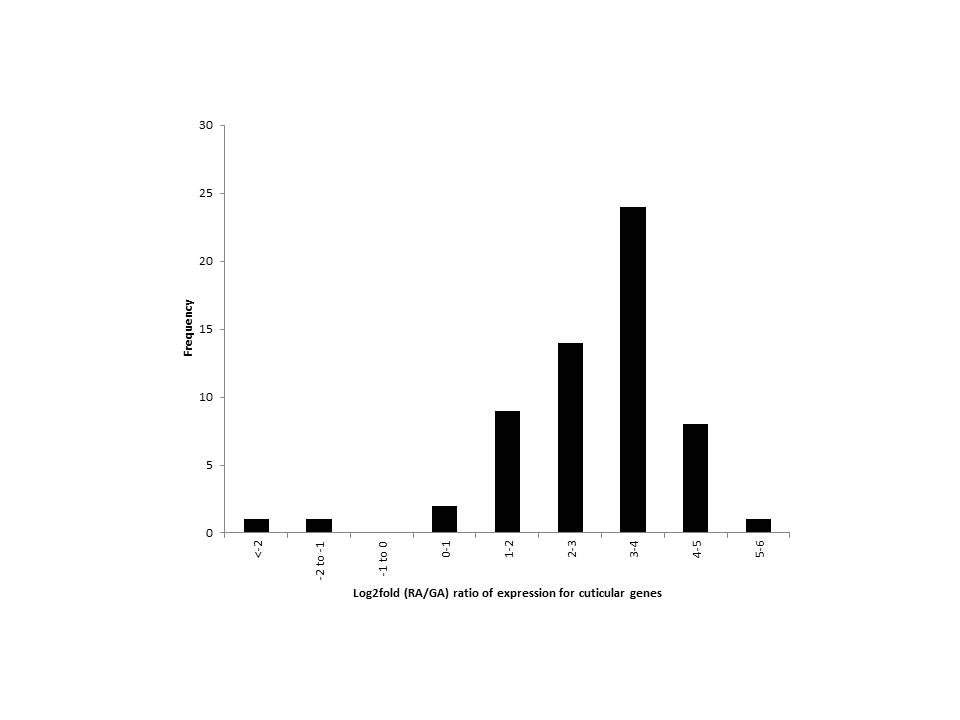

Supplement: Additional file 4: — Distribution of log2fold ratios of expression (RA/GA) for 60 contigs annotated as “cuticular protein”. (JPG 18 kb) [file 12864_2016_2530_MOESM4_ESM.jpg]
